# The role of serum C-reactive protein in the diagnosis of periprosthetic shoulder infection

**DOI:** 10.1007/s00402-021-03779-2

**Published:** 2021-01-30

**Authors:** Doruk Akgün, Mats Wiethölter, Paul Siegert, Victor Danzinger, Marvin Minkus, Karl Friedrich Braun, Philipp Moroder

**Affiliations:** Department for Shoulder and Elbow Surgery, Center for Musculoskeletal Surgery, Humboldt-Universität zu Berlin, and Berlin Institute of Health, Charité–University Medicine Berlin, Corporate Member of Freie Universität Berlin, Augustenburger Platz 1, 13353 Berlin, Germany

**Keywords:** C-reactive protein, Periprosthetic shoulder infection, Definition, Low-virulence

## Abstract

**Introduction:**

There is a paucity of literature regarding serum C-reactive protein (CRP) in the evaluation of a shoulder periprosthetic joint infection (PJI). The purpose of the current study was to establish cutoff values for diagnosing shoulder PJI and evaluate the influence of the type of infecting microorganism and the classification subgroups according to last proposed International Consensus Meeting (ICM) criteria on the CRP level.

**Materials and methods:**

A retrospective analysis of all 136 patients, who underwent septic or aseptic revision shoulder arthroplasty in our institution between January 2010 and December 2019, was performed. Shoulder PJI was defined according to the last proposed definition criteria of the ICM. Serum CRP levels were compared between infected and non-infected cases, between infection subgroups, as well as between different species of infecting microorganisms. A receiver-operating characteristic (ROC) analysis was performed to display sensitivity and specificity of serum CRP level for shoulder PJI.

**Results:**

A total of 52 patients (38%) were classified as infected, 18 meeting the criteria for definitive infection, 26 for probable infection and 8 for possible infection. According to the ROC curve, an optimized serum CRP threshold of 7.2 mg/l had a sensitivity of 69% and specificity of 74% (area under curve = 0.72). Patients with definitive infection group demonstrated significantly higher median serum CRP levels (24.3 mg/l), when compared to probable, possible infection groups and PJI unlikely group (8 mg/l, 8.3 mg/l, 3.6 mg/l, respectively, *p* < 0.05). The most common isolated microorganism was *Cutibacterium acnes* in 25 patients (48%) followed by coagulase-negative staphylococci (CNS) in 20 patients (39%). Patients with a PJI caused by high-virulent microorganisms had a significantly higher median serum CRP level compared to patients with PJI caused by low-virulent microorganisms (48 mg/l vs. 11.3 mg/l, *p* = 0.04).

**Conclusions:**

Serum CRP showed a low sensitivity and specificity for the diagnosis of shoulder PJI, even applying cutoffs optimized by receiver-operating curve analysis. Low-virulent microorganisms and patients with probable and possible infections are associated with lower CRP levels compared to patients with definitive infection and infections caused by high-virulent microorganisms.

**Level of evidence:**

Diagnostic Level III.

## Introduction

The accurate diagnosis of shoulder periprosthetic joint infection (PJI) continues to pose a difficult task due to the subtle clinical presentation of common low-grade infections, especially involving *Cutibacterium acnes*, which is identified in both primary and revision shoulder arthroplasties in up to 70% of the cases with positive cultures [1–3]. Although acute infections are readily detectable mostly due to clinical symptoms and high levels of inflammatory biomarkers, chronic low-grade infections present a unique diagnostic and therapeutic challenge. C-reactive protein (CRP) has been shown to be a powerful parameter for detecting periprosthetic joint infections and recommended by the American Academy of Orthopaedic Surgeons (AAOS) [4] and the International Consensus Meeting (ICM) [5] as first-line test in the diagnosis of PJI. However, recent literature showed high false-negative rates, especially in patients with low-virulent microorganisms [6–9].

There is a paucity of literature regarding serum CRP in the evaluation of shoulder PJI and existing studies show sensitivities varying between 30 and 91% [10–14]. These differences in sensitivities between studies can be related to the different definitions criteria used in these studies and different types of bacteria. Recently, new criteria was proposed for shoulder PJI classifying it into 4 subgroups; (1) definitive infection, (2) probable infection, (3) possible infection and (4) infection unlikely [15]. This stratification allows for more homogenous groups with different characteristics of PJI and levels of inflammation. The purpose of the current study was to establish cutoff values for diagnosing shoulder PJI and evaluate the influence of the type of infecting microorganism and the classification subgroups on the CRP level.

## Materials and methods

A retrospective analysis of all patients, who underwent septic or aseptic revision shoulder arthroplasty in our institution between January 2010 and December 2019 was performed. Following data were recorded for each patient: gender and age, involved joint, clinical symptoms, surgical history of the involved joint, type of arthroplasty, concurrent antibiotic treatment, as well as radiological assessment. The standard diagnostic protocol to identify PJI included the following assessments in all patients; Laboratory values including C-reactive protein, serum leucocyte count as well as results of preoperative aspiration, if performed, including leucocyte count, neutrophil percentage, microbiologic and histopathologic results, radiological and intraoperative evaluation of the component loosening and intraoperative findings, such as cloudy fluid or gross intra-articular purulence and microbiological and pathological results of revision surgery. Furthermore, the total number of intraoperative tissue samples and the incidence of positive cultures were recorded for each patient. Patients with less than two tissue specimens for culture were excluded. Sonication fluid cultures were incorporated into the infection criteria as microbiologic results used for the infection definition.

Specimens for microbiological analysis were collected with a new sterile instrument each time, were placed directly into sterile containers without touching by hand and sent immediately with retrieved implants to our microbiology laboratory for further analysis within 1 h after surgery. The microbiologic specimen as well as sonication fluid cultures were placed onto aerobic and anaerobic sheep blood agar plates and incubated for 14 days. Sonication was performed as previously described in every case after January 2014 [16]. Shoulder PJI was diagnosed according to the last proposed definition criteria of the ICM and based on this definition CRP levels of greater than 10 mg/l were considered elevated [15]. According to these criteria, patients were classified into 4 infection subgroups: (1) definitive infection, (2) probable infection, (3) possible infection and (4) infection unlikely. Meeting one of the following criteria was diagnostic of definitive periprosthetic shoulder infection: (1) a sinus tract communicating with the prosthesis; (2) gross intra-articular pus; (3) two positive cultures with phenotypically identical virulent organisms. In the lack of these defining signs, weighted minor criteria (Table 1) are summed and used to distinguish between probable, possible and unlikely infection.

**Table 1:** Minor criteria

	Weight
Unexpected wound drainage	4
Single positive tissue culture (virulent organism)	3
Single positive tissue culture (low-virulence organism)	1
Second positive tissue culture (identical low-virulence organism)	3
Humeral loosening	3
Positive frozen section (5 PMN in at least 5 high-power fields)	3
Positive preoperative aspirate culture (low or high virulence)	3
Elevated synovial neutrophil percentage (> 80%)	2
Elevated synovial WBC (> 3000 cells/µl)	2
Elevated ESR (> 30 mm/h)	2
Elevated CRP (> 10 mg/l)	2
Cloudy fluid	2

The three categories in these less distinct scenarios are defined as follows:

- Six or greater with identified organism: probable infection.
- Six or greater without identified organism: possible infection.
- Fewer than six.
  - Single positive culture virulent organism: possible infection.
  - Two positive culture low-virulence organisms: possible infection.
- Negative cultures or only single positive culture for low-virulent organism: infection unlikely [15, 17].

### Statistical analysis

Chi-squared and Fisher’s exact tests were used to find significant differences between categorical variables. The two-sample *t* test (for parametric distribution) or Mann–Whitney *U* test (for non-parametric distribution) was used to compare continuous variables between the groups. Median serum CRP levels were calculated for various species of microorganisms [*S. aureus*, *C. acnes*, coagulase-negative staphylococci (CNS)] and for definitive, probable, possible infection and infection unlikely groups. Groups were then compared to each other for a statistically significant difference using Kruskal–Wallis test adjusting for multiple comparisons with Dunn’s test. When analyzing species-specific results, monomicrobial and polymicrobial infections with only the same species were compared. For a further analysis of the diagnostic utility of serum CRP in all patients, patients in the definitive, probable and possible infection groups were combined and defined as infection group and patients in the infection unlikely group were defined as non-infection group. A receiver-operating characteristic (ROC) analysis was performed to display sensitivity and specificity of serum CRP level for shoulder PJI. The area under the curve (AUC) was calculated and the optimum cutoff point was determined using the maximized Youden’s index. The results were expressed as mean and standard deviation (SD) or as number and percentage. A *p* value < 0.05 was considered significant. SPSS version 20 (SPSS Inc., Chicago, Illinois) was used for the statistical analyses.

## Results

One hundred and thirty-six patients were included in this study. The mean age and standard deviation (SD) of the patients were 70 ± 10.3 years (range 37–91) and 79 patients were females (58%). A total of 10 patients (7%) had undergone at least 1 previous septic revision, 24 (18%) had undergone an aseptic revision and 102 (75%) had developed a PJI after the initial arthroplasty. The median interval from last surgical treatment until the revision surgery at our institution was 18.6 months (0.7–166). The type of arthroplasty at the time of revision surgery was hemiarthroplasty in 48 patients, total shoulder arthroplasty in 38 and reverse shoulder arthroplasty in 50 patients. Besides infection, the reasons for revision surgery included loosening of the components, overstuffing, secondary rotator cuff insufficiency and instability.

A total of 52 patients (38%) were classified as infected, 18 meeting the criteria for definitive infection, 26 for probable infection and 8 for possible infection. In 84 (62%) patients, the infection was considered unlikely. A total of 560 cultures were taken in all patients and 142 (25%) were positive. A polymicrobial infection was identified in 13 patients (25%). Further demographic and clinical characteristics of the patients are summarized in Table 2.

**Table 2:** Patient demographics and CRP findings

Variable	All patients, *n* = 136	Infection group, *n* = 52	Non-infection group, *n* = 84	*p* value
Mean age, year^a^	70 ± 10.3	70.6 ± 10	69.6 ± 10.4	0.6
Gender^b^				
Male	57 (42)	27 (52)	30 (36)	0.08
Female	79 (58)	25 (48)	54 (64)	
CRP at admission (mg/l)^a^	14.7 ± 27	27 ± 39	7.1 ± 11	0.0001

^a^The values are given as the mean and the standard deviation

^b^The values are given as the number with the percentage of the group in parentheses

**Fig. 1:**
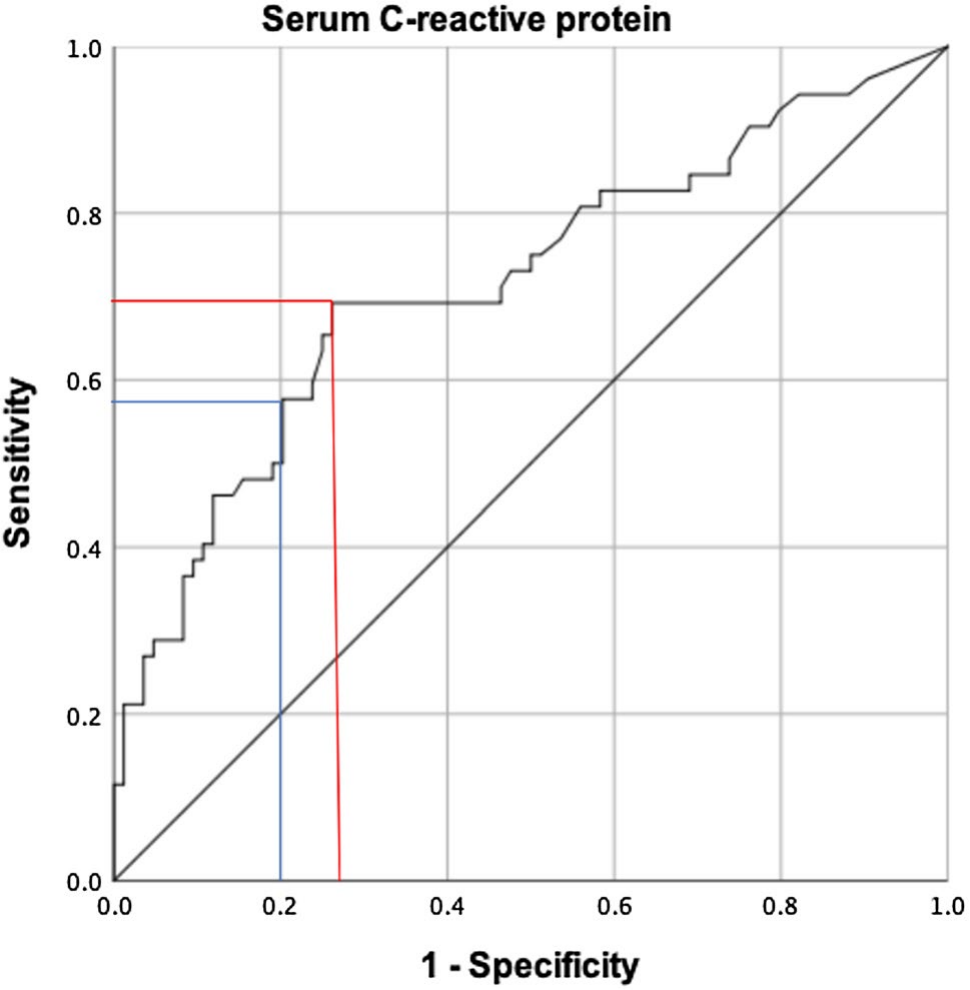
Receiver-operating characteristics curve for serum C-reactive protein. Sensitivity is plotted against 100% specificity. The red and blue lines mark the sensitivity and 1-specificity for the optimized threshold (Youden’s index) of 7.2 mg/l and ICM definition cutoff of 10 mg/l, respectively

There was a significant difference in serum CRP levels between infection and non-infection groups (27 mg/l vs. 7.1 mg/l, *p* < 0.001). A serum CRP threshold of 10 mg/l had a sensitivity of 58% and a specificity of 80%. According to the ROC curve, an optimized serum CRP threshold of 7.2 mg/l had a sensitivity of 69% and specificity of 74% (AUC = 0.72) (Fig. 1). Twenty-two patients (42.3%) from the infection group had a normal (< 10 mg/l) serum CRP level prior to revision surgery. When a diagnostic threshold of 7.2 mg/l is used, 16 patients (31%) had still a false-negative CRP result.

**Fig. 2:**
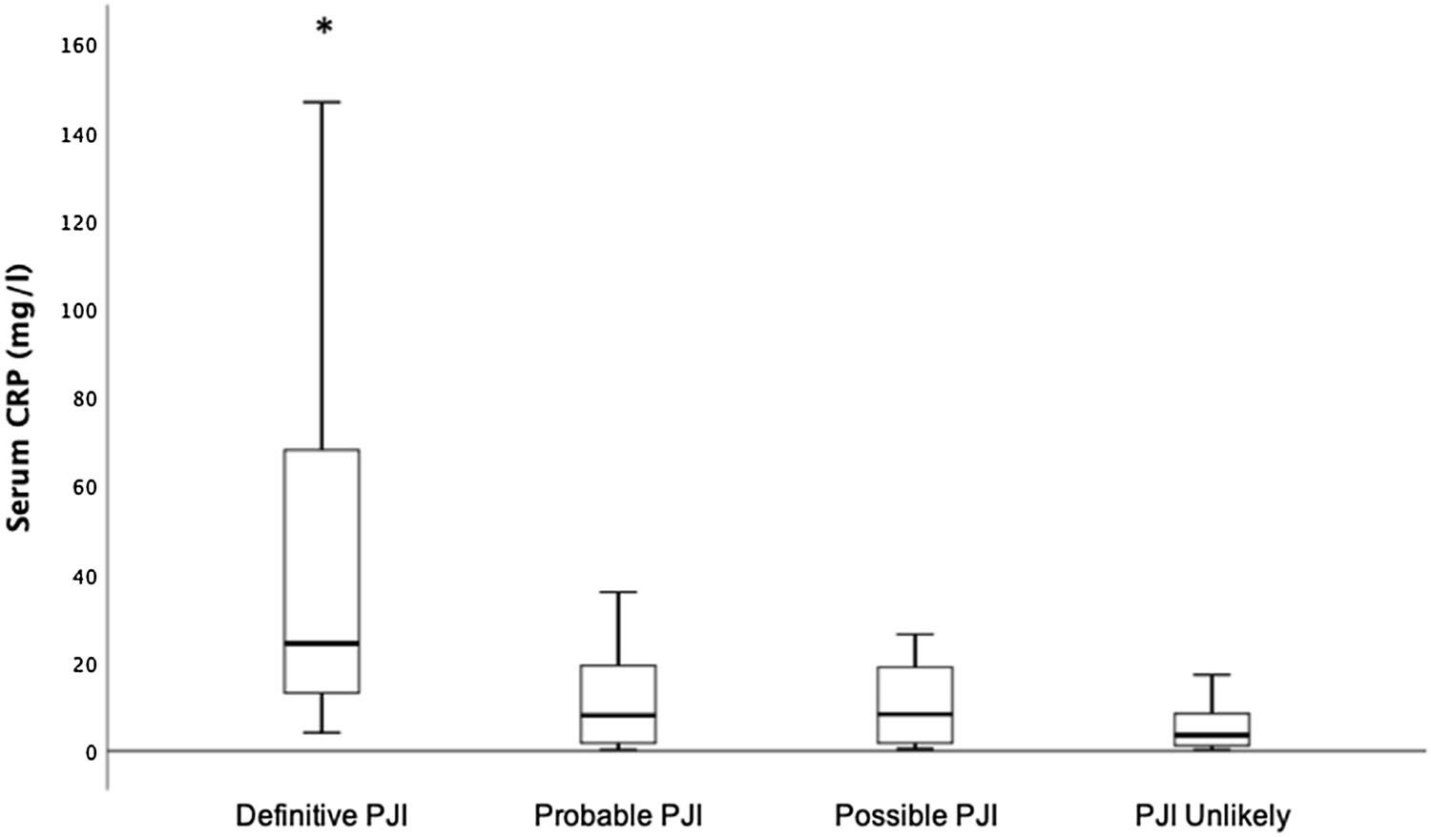
Box-and-whisker plot showing dependency of serum C-reactive protein (CRP) level on the different periprosthetic joint infection (PJI) classification subgroups. The horizontal black line marks the median CRP level; the box, the interquartile range; and the whiskers, the minimum and maximum. Asterisks indicate groups with significantly higher median CRP levels compared to groups without asterisks

When grouping infected patients by infection subgroups, patients in the definitive infection group demonstrated significantly higher median serum CRP levels (24.3 mg/l), when compared to probable, possible infection groups and PJI unlikely group (8 mg/l, 8.3 mg/l, 3.6 mg/l, respectively, *p* < 0.05) (Fig. 2). While 16 of 26 patients (62%) with probable infection, 5 of 8 patients (63%) with possible infection and 71 of 84 patients (84%) without shoulder PJI had a normal (< 10 mg/l) serum CRP level, only 1 of 18 patients (6%) with definitive infection had a normal CRP level. Interestingly, patients with normal preoperative CRP had similar rates of positive cultures compared to patients with elevated CRP (46/96 (48%) vs. 78/137 (57%), *p* = 0.2).

**Fig. 3:**
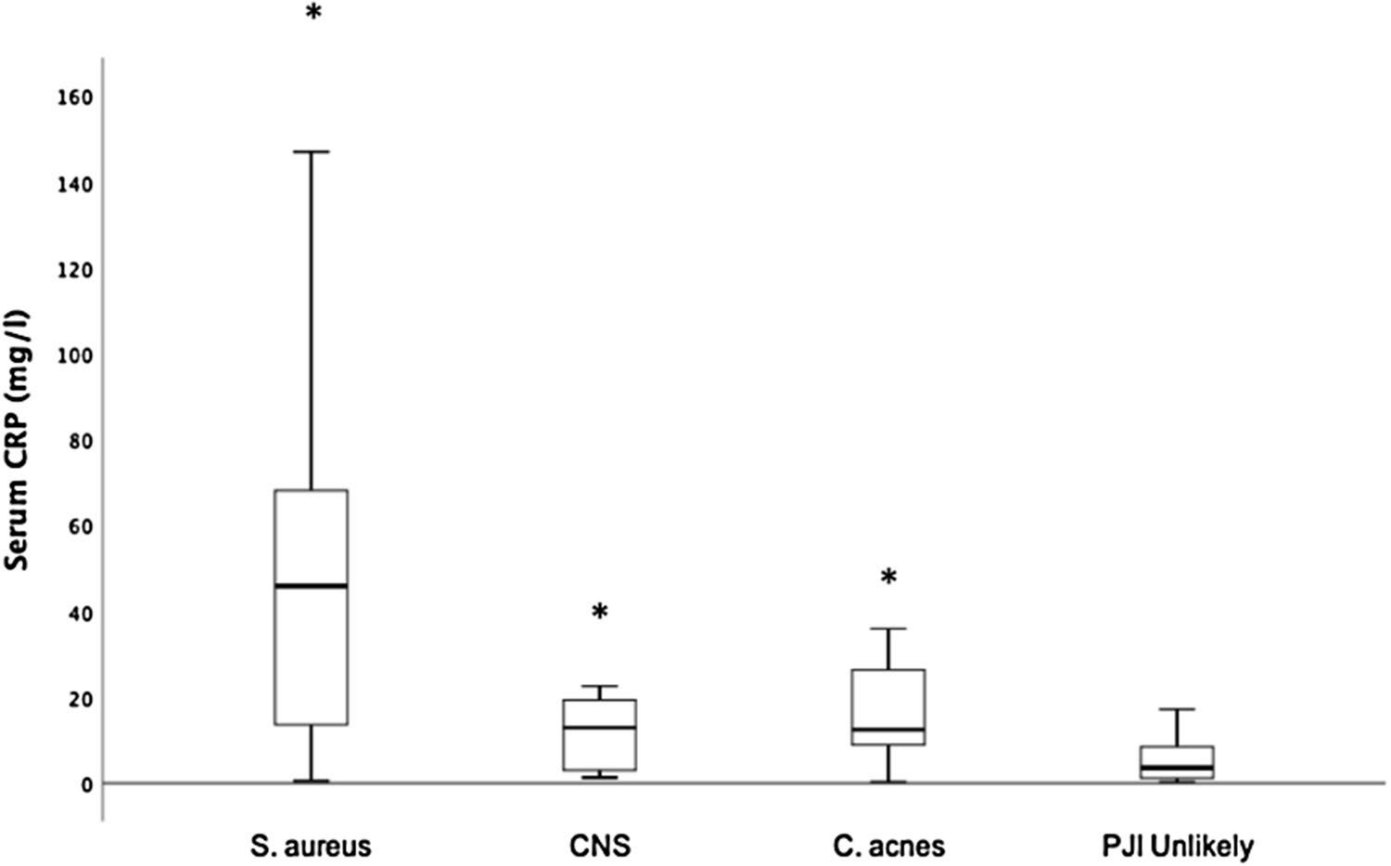
Box-and-whisker plot showing dependency of serum C-reactive protein (CRP) level on the infecting microorganism and patients with unlikely periprosthetic joint infection (PJI). The horizontal black line marks the median CRP level; the box, the interquartile range; and the whiskers, the minimum and maximum. Asterisks indicate groups with significantly higher median CRP levels compared to groups without asterisks. *Staphylococcus aureus*, *n* = 5; coagulase-negative staphylococci (CNS), *n* = 10; *Cutibacterium acnes*, *n* = 14; infection unlikely, *n* = 84

**Fig. 4:**
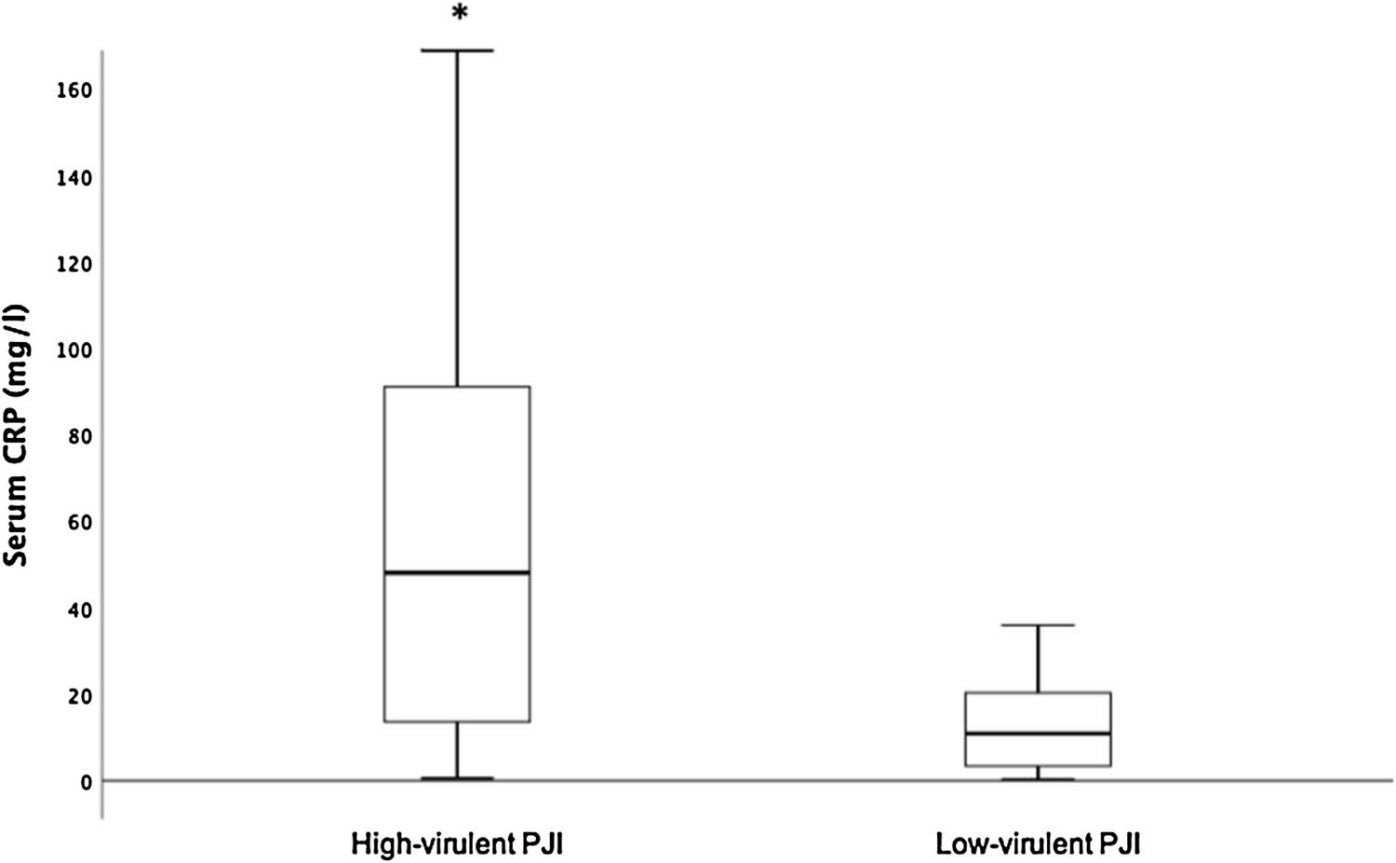
Box-and-whisker plot showing dependency of serum C-reactive protein (CRP) level on the virulence of periprosthetic joint infection (PJI). The horizontal black line marks the median CRP level; the box, the interquartile range; and the whiskers, the minimum and maximum. Asterisks indicate groups with significantly higher median CRP levels compared to groups without asterisks. High-virulent PJI, *n* = 10; low-virulent PJI, *n* = 36

The most common isolated microorganism was *Cutibacterium acnes* in 25 patients (48%) followed by CNS in 20 patients (39%). *Staphylococcus aureus* was isolated in 6 patients with definitive infection and in 2 patients with probable infection. Table 3 summarizes the causative pathogens. Patients with *C. acnes* and/or CNS infection represented 73% of all shoulder PJIs in patients with a normal CRP level. When grouping microorganisms by species, patients with *S. aureus*, CNS and *C. acnes* had a significantly higher median serum CRP levels (46 mg/l, 12.9 mg/l and 12.5 mg/l, respectively) compared with patients with unlikely PJI (3.6 mg/l, *p* < 0.05). Although the patients with S. aureus had a higher median serum CRP level compared to patients with CNS and *C. acnes*, this difference was not statistically significant (Fig. 3). Furthermore, patients with a PJI caused by high-virulent microorganisms had a higher median serum CRP level compared to patients with PJI caused by low-virulent microorganisms (48 mg/l vs. 11.3 mg/l, *p* = 0.04) (Fig. 4).

**Table 3:** Microorganisms identified in patients with PJI

Microorganisms	No of patients with PJI, *n* = 52 (%)
*Cutibacterium acnes*	25 (48)
Coagulase-negative staphylococci	20 (39)
*Staphylococcus aureus*	8 (15)
*Enterococcus faecalis*	2 (4)
*Streptococcus* spp.	2 (4)
Others	4 (8)
Polymicrobial	13 (25)
Negative microbiology	6 (12)

## Discussion

Serum CRP has been an important biomarker for the diagnosis of periprosthetic infection for a long time. However, recent studies were able to show a high false-negative rate in patients with PJI, especially infected by low-virulent microorganisms [6–9]. Furthermore, there is still a paucity of literature regarding serum CRP in the evaluation of a shoulder PJI. In the last proposed ICM definition of shoulder PJI, an elevated serum CRP level was defined as a minor criteria and the threshold was determined as 10 mg/l according to the hip and knee PJI literature [15, 18]. However, our study demonstrated a low sensitivity and specificity of 58% and 80%, respectively, of serum CRP threshold of 10 mg/l. Furthermore, we were able to find a more optimized threshold of 7.2 mg/l with a sensitivity of 69% and specificity of 74%. While missing almost half of the patients with a shoulder PJI if a cutoff value of 10 mg/l is applied, only one-third of the patients had a false-negative serum CRP level prior to revision surgery, if a diagnostic threshold of 7.2 mg/l is used.

Piper et al. have investigated the role of serum CRP in hip, knee and shoulder PJI, as well as in patients with spinal implant infections [14]. They found out, that CRP showed the lowest sensitivity for the diagnosis of shoulder PJI, even applying cutoffs optimized using receiving operating curve analysis. The optimized CRP cutoff was 7 mg/l with a sensitivity of 63% and specificity of 73%, which is similar to the results of the current study. Other studies reported sensitivities of CRP varying between 30 and 91% [10–12]. In a recent meta-analysis, Nelson et al. reported, that 62.1% of patients, who were treated for shoulder PJI had an elevated CRP level [13].

The high prevalence of low-virulent microorganisms in CRP-negative PJIs observed in our study had previously been reported by the other authors [19–21]. In a study by McArthur, low-virulent microorganisms were significantly more often associated with normal CRP results compared to high-virulent microorganisms. Obvious clinical signs of infection such as redness, swelling, sinus tract formation and fever are rarely encountered in infections caused by these low-virulent microorganisms and especially in these cases, CRP should not be used alone to rule out a diagnosis of shoulder PJI [22]. Our results also support the hypothesis that CRP response can be weak or even non-existent in patients with PJI caused by low-virulent microorganisms. This difference can be attributed to the fact that high-virulent microorganisms cause an acute planktonic infection inducing a high inflammation with release of cytokines and elevation of CRP, while low-virulent microorganisms adhere rapidly to implant surfaces forming biofilms, where they escape from the host immune system, which results in reduced inflammation [6, 23, 24]. This is especially true for *C. acnes*, which is a Gram-positive slow-growing microorganism. It can isolate itself from the host defense by forming a biofilm, which makes detection and complete eradication difficult [25]. Similar to the literature, *C. acnes* was also the most common isolated microorganism in our cohort affecting almost half of the patients with shoulder PJI, followed by coagulase-negative staphylococci. Accordingly, low-virulent microorganisms represented almost 75% of all shoulder PJIs in patients with a normal CRP level.

Given the lack of strong evidence defining the clinical relevance of low-grade infections, new ICM definition criteria divided shoulder PJI into 4 subgroups, which allows for more homogenous groups with different characteristics of PJI and level of inflammation. In our study, patients with a definitive infection showed significantly higher serum CRP levels compared to patients with probable or possible infections. While mostly high-virulent microorganisms were evident in patients with definitive infection, such as *Staphylococcus aureus* or streptococci, interestingly four patients had an infection with *C. acnes*. One patient had a polymicrobial infection with *C. acnes* and *Staphylococcus aureus*, whereas the remaining three patients had a monomicrobial infection with *C. acnes*. These patients were classified as definitively infected due to the presence of a sinus tract or gross intra-articular pus. This difference in pathogenic potential and level of host inflammatory response might be caused by different phylotypes of *C. acnes* with varying virulence properties [26–28]. Boyle et al. were recently able to show that hemolytic strains of *C. acnes* exhibit enhanced pathogenicity to their host by eliciting a more prominent systemic inflammatory response and severe clinical course [26]. Furthermore, they reported a significantly higher median serum CRP level (17.9 mg/l) in the hemolytic *C*. *acnes* group compared to the nonhemolytic group (3.5 mg/l). Thus, CRP seems to be a more sensitive serum marker for inflammation than erythrocyte sedimentation rate and blood leucocyte count.

This study has limitations. First, we did not exclude patients with inflammatory diseases, which can alter our results. However, it was shown in the literature that there is no difference in the threshold of CRP level used to diagnose PJI in patients with or without an inflammatory disease [5]. Second, it was a retrospective analysis with lack of every available data for each patient. Third, despite using the last proposed definition criteria for shoulder PJI, some patients may be misdiagnosed as infected and vice versa.

In conclusion, serum CRP showed a low sensitivity and specificity for the diagnosis of shoulder PJI, even when applying cutoffs optimized using receiver-operating curve analysis. Low-virulent microorganisms and patients with probable and possible infections are associated with lower CRP levels compared to patients with definitive infection and infections caused by high-virulent microorganisms.
